# Starving or Stuffing? Plasticity in Wild Boar Body Mass Variations During Summer in a Mediterranean Area

**DOI:** 10.1111/1749-4877.13012

**Published:** 2025-08-06

**Authors:** Martina Calosi, Niccolò Fattorini, Andrea Sforzi, Luca Tonini, Francesco Ferretti

**Affiliations:** ^1^ Research Unit of Behavioural Ecology, Ethology and Wildlife Management, Department of Life Sciences University of Siena Siena Italy; ^2^ NBFC, National Biodiversity Future Center Palermo Italy; ^3^ Maremma Natural History Museum Grosseto Italy; ^4^ Maremma Regional Park Agency Grosseto Italy

**Keywords:** crops, density, ecotones, monitoring, swine, *Sus scrofa*, wild pigs

## Abstract

Identifying determinants of key phenotypic indicators driving animal population dynamics is fundamental to address measures aimed at mitigating human–wildlife interactions. In Mediterranean areas, summer drought reduces the availability of water and food resources for wild ungulates, potentially affecting body conditions. Since summer is a crucial period for the nursing/weaning of offspring, a seasonal bottleneck for ungulates would be expected to occur, especially in females. However, data on fluctuations of ungulate body mass during summer are scarce. We investigated the effects of summer progression, aridity, population density, and land use on body mass variation of adult/yearling wild boar (*Sus scrofa*) in a mixed forested‐rural Mediterranean protected area, over a 16‐year period (2007–2022). Data were gathered from individuals harvested during population control management actions, in summer. In contrast with our predictions, no consistent mass loss was observed throughout the summer. According to expectations, body mass was favored by decreasing aridity in the previous 3 months. In males, the positive effect of rainfall was mitigated by population density. Females experienced mass gain in non‐arid years, mass loss in arid years, and no change in “average” years. The importance of edge habitats located at the interface between wooded and cultivated areas was confirmed by a positive relationship between female body mass and ecotone availability. The results indicate that, in a mixed forested‐rural area, wild boar could maintain, or even increase, their body mass during summer. Moreover, findings emphasize the crucial role of rainfall in modulating a key driver of population dynamics for this ungulate in Mediterranean areas.

## Graphical abstract

Assessing drivers of population dynamics helps to mitigate human–wildlife conflicts. In Mediterranean areas, summer aridity reduces resources, potentially affecting ungulates' body condition and reproduction. In a mixed forested‐rural area, wild boar female body mass increased throughout summer in rainy‐mild years and decreased in hot‐dry years. In males, the mass gain was hindered by aridity and decreased with higher population densities. Occasional use of cultivated crops may buffer against summer mass loss.

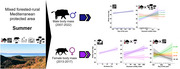

## 1 Introduction

Handling human–wildlife interactions is increasingly important in the current landscape and ecosystem management. Identifying and monitoring key indicators of population dynamics is fundamental for collecting basic information and defining actions aiming at the mitigation of the ensuing potential conflicts, especially in anthropized ecosystems. Particular attention in this respect should be given to indicators of individual performance such as morphometric traits (Lashley et al. 2015; Risco et al. 2018; Vannini et al. 2021; Baruzzi et al. 2023). Among these traits, body mass is a key driver of individual health, survival, and reproductive potential which, in turn, drives population dynamics (Ozgul et al. 2010; Albon et al. 2017; Risco et al. 2018). In mammals, environmental fluctuations and variations in population densities are pivotal in influencing individual mass (Ozgul et al. 2010; English et al. 2014), and their monitoring helps to identify animal responses to environmental changes. In particular, there is an increasing interest in assessing animal responses in agricultural landscapes (e.g., Hewison and Gaillard 2001; Vannini et al. 2021; Gethöffer et al. 2023), where potential conflicts with agricultural activities can arise.

Ungulates represent a group of mammals distributed worldwide (except Antarctica; Wilson and Mittermeier 2011), with great relevance for the management of human–wildlife interactions (e.g., Putman and Moore 1998; Barrios‐Garcia and Ballari 2012; Carpio et al. 2021). They can survive in a wide range of habitats and climates and can adapt some of their morphological characteristics, such as body mass, to local conditions (Geisser and Reyer 2005; Toїgo et al. 2006). Variations in food availability and quality can influence body mass and size, with larger and heavier individuals usually associated with habitats with plenty of resources, where the amount of food supply allows them to invest in body growth and to store fat reserves (e.g., Power 1983; Hinsley et al. 2008; Rughetti and Festa‐Bianchet 2012). Population density is a body size regulator, with smaller individuals usually associated with higher densities, in response to stronger competition for food (Morellet et al. 2007). Climatic fluctuations also influence resource cycles such as plant development or water availability, thus acting as drivers of individual growth, health, and reproductive success (Geisser and Reyer 2005; Toїgo et al. 2006; Frauendorf et al. 2016; Vannini et al. 2021). Spring precipitation may positively affect ungulate density, with higher densities associated with higher precipitation during the spring months in Mediterranean landscapes (Colomer et al. 2024), consequently affecting individual growth (Morellet et al. 2007).

The influence of climatic conditions may be particularly effective in regions characterized by harsh seasons, where drought occurs seasonally (Giralt‐Rueda and Santamaría 2023). Mediterranean regions are characterized by mild winters and hot‐dry summers. In these areas, the annual rainfall is generally scarce, mainly concentrated in autumn and spring, while summer represents the most limiting season, when the scarcity of food resources may affect ungulate densities and/or individual body mass (Massei et al. 1997; Fernández‐Llario and Carranza 2000; Giralt‐Rueda and Santamaría 2023). Thus, the most limiting season tends to overlap with crucial periods of animal life cycles, including nursing, weaning, and early offspring growth. Data on ungulate body mass are generally scarce for summer because they are usually collected during the hunting season, i.e., mostly in late summer‐early winter (Europe: Apollonio et al. 2011; Africa: Crosmary et al. 2012; North America: Little et al. 2014; Oceania: Forsyth et al. 2014).

Our aim was to investigate the main drivers affecting wild boar (*Sus scrofa*) body mass during summer in a Mediterranean area. We performed sex‐based analyses to assess how the absolute body mass of individuals varied as summer progressed, while accounting for variations in weather conditions, population density, and land use. Our analysis was based on individuals harvested during population control actions over a 16‐year period.

Wild boar is the most widespread ungulate at the global scale, and its recent increase in numbers, density, and distribution is triggering significant worries about the potential negative impacts on the conservation of threatened habitats/species, as well as on human activities (Ballari and Barrios‐García 2014; Castillo‐Contreras et al. 2018; Markov et al. 2019; Barasona et al. 2021). This suid is omnivorous and adapts its diet switching between above and underground resources, following variations in food availability (Herrero et al. 2006; Ballari and Barrios‐Garcia 2014; Laguna et al. 2021). Harsh climatic conditions such as prolonged droughts may limit their foraging activity by making the soil harder to dig and/or leading to a reduction of vegetation and soil productivity (Yokoyama et al. 2020; Ruf et al. 2023; Yang et al. 2024; Calosi et al. 2024). Consequently, these conditions could trigger a decrease in individual body mass, affecting reproductive success and survival (Massei et al. 1997, 1996; Fernández‐Llario et al. 1999; Geisser and Reyer 2005; Frauendorf et al. 2016; Risco et al. 2018; Brogi et al. 2023). These effects may have a significant negative impact, particularly on females, due to the high energy demands of gestation, parturition, and lactation (Ruf et al. 2021). In Mediterranean landscapes, the first two phases mostly overlap with the peak of vegetation growth (i.e., spring; Fernández‐Llario and Carranza 2000; Briedermann 2009). Conversely, lactation usually persists for at least 3 months after birth (Apollonio et al. 2011). It may still occur in summer, when the availability of natural food resources is minimal, requiring the exploitation of fat reserves to supply energy demands (Mauget 1982). Thus, drought and scarcity of natural food resources during summer are expected to affect individual body mass, emphasizing the costs of lactation for females, as well as reducing individual survival and reproductive potential in the population (Massei et al. 1996; Fernández‐Llario et al. 1999; Geisser and Reyer 2005; Frauendorf et al. 2016; Brogi et al. 2023). In addition, female conditions could affect piglets' survival, either through decreased lactation or through exposure to the same food shortage conditions, with effects on population dynamics (González‐Crespo et al. 2018). In agricultural landscapes, crops can provide a high‐quality food supply during harsh seasons, increasing individual survival and maintenance of body mass, also triggering conflicts with farmers (Herrero et al. 2006; Vetter et al. 2020; Pascual‐Rico et al. 2021; Gethöffer et al. 2023; Ruf et al. 2023; Yang et al. 2024). Wild boar tend to optimize shelter and resource availability by selecting habitats that ensure both energetic supply and avoidance of disturbance, such as ecotones close to both shrubwood and cultivated crops (Saito et al. 2012; Cappa et al. 2019; Laguna et al. 2021). This space‐use strategy could be particularly effective in Mediterranean landscapes during summer, where food resources are mainly concentrated in transitional habitats between forests and open areas, as well as in meadows and grasslands, rather than in drier habitats such as sclerophyllic shrubland (Massei et al. 1996; Spitz et al. 1998; Torres‐Porras et al. 2015; Fattorini and Ferretti 2020).

We concentrated on the effects on body mass potentially triggered by (*i*) seasonal progression of summer, while still considering the potential effects of (*ii*) aridity, and (*iii*) habitat type. We expected (1) a decrease in wild boar body mass throughout summer. We predicted such a decrease be stronger for both sexes (1a) in drier years, (1b) in years when population density is higher, and, for females, (1c) especially in adult individuals, which are expected to face the harsh season with dependent offspring more frequently than yearling ones. Moreover, we expected (2) an increase in summer body mass with decreasing aridity in the previous months, that is, lower body masses in arid years (Massei et al. 1997, 1996). Eventually, we expected (3) an increase in summer body mass for individuals with a greater availability of transitional habitats and crops in their home ranges, due to higher food availability in these habitats, compared to the “poorer” Mediterranean shrubland.

## 2 Materials and Methods

### 2.1 Study Area

**FIGURE 1:**
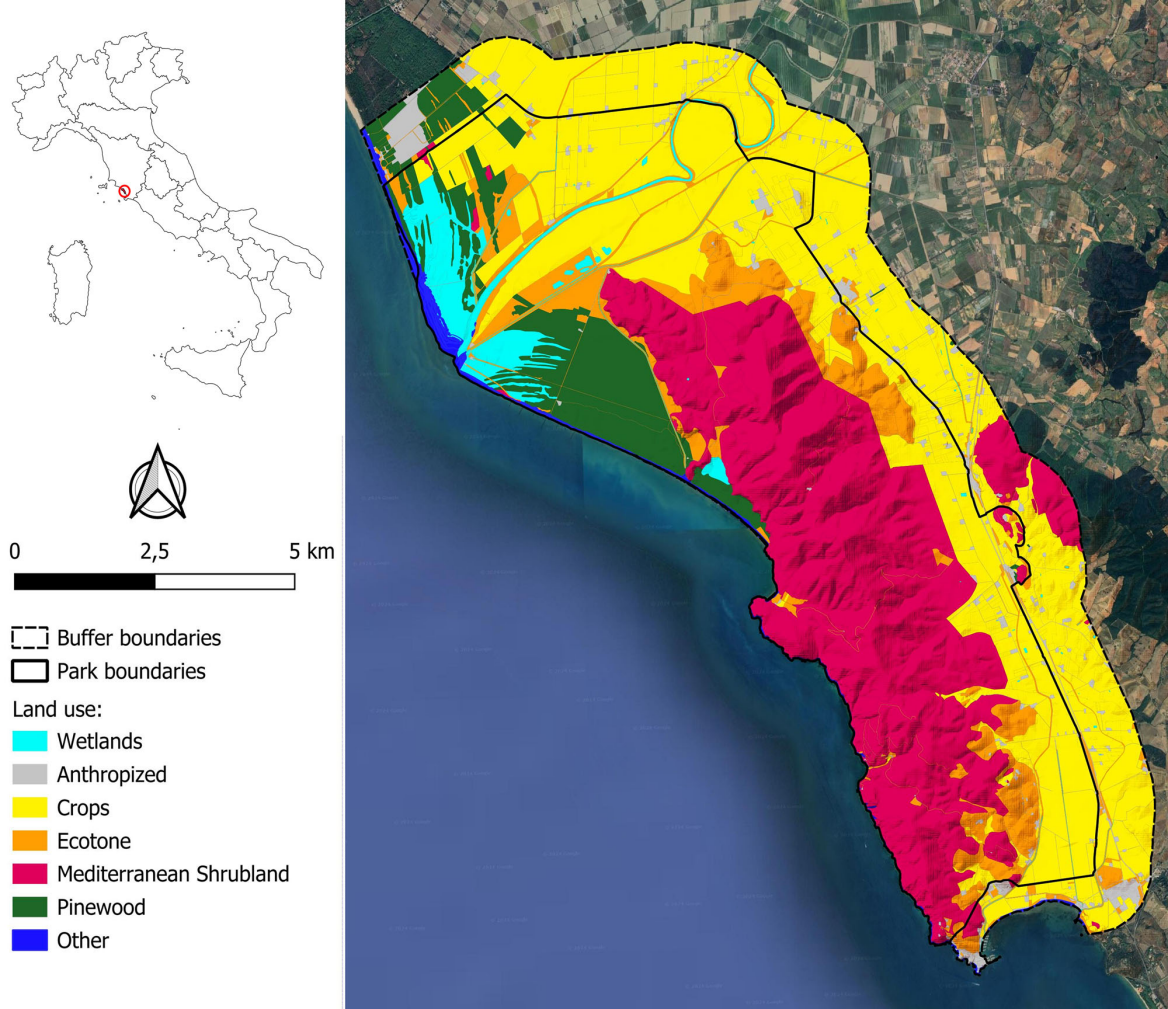
Study area. Location of Maremma Regional Park and land use within the Park boundaries (black solid contour) and a buffer zone of 1.1 km around the Park boundaries (black dotted contour).

The study was carried out in the Maremma Regional Park, in central Italy (MRP; centroid's coordinates: Lat. 42.641613°N, Long. 11.088434°E; extent: ∼90 km^2^; elevation: 0–417 m a.s.l.; Figure 1). This coastal protected area is characterized by a Mediterranean climate, with mild winter and hot‐dry summer. Rainfall is concentrated from autumn to spring (seasonal averages of min–max temperatures and rainfall in the study period; autumn: 10–19°C, ∼206 mm; winter: 4–14°C, ∼125 mm; spring: 11–21°C, ∼99 mm; summer: 17–29°C, ∼ 62 mm; data from Servizio Idrologico Regione Toscana). The area is mainly covered by Mediterranean shrubland (40%), including oakwood and scrubland/garigue, dominated by sclerophyllic plant species such as *Quercus ilex*, *Juniperus* spp., *Erica* spp., *Myrtus communis*, and *Phyllirea* spp. The northwest sector includes a pinewood dominated by stone pine (*Pinus pinea*) (9%) and wetlands (5%). The landscape is also covered by crops (30%; mainly wheat, cereals, and sunflowers in summer, locally irrigated) and habitats that we termed “ecotones” composed of open meadows, set‐aside grasslands, and pastures, including olive groves partially abandoned and recolonized by shrubwood (13%). The remaining area is covered by human settlements (2%) and other habitats (mostly seaside, 1%). Wild boar density ranged from ∼10 to ∼30 individuals/km^2^ throughout the study period (Ferretti et al. 2016, 2023; Fattorini and Ferretti 2020), with a reduction from 2010 to 2018, and subsequent stability, due to increased population control operated by the Park Agency (Ferretti et al. 2016; Fattorini and Ferretti 2020). A previous study on habitat use inferred from fecal counts and signs of presence (rooting activity) showed that, in summer, the wild boar uses ecotones significantly more than other habitat types in the study area (Fattorini and Ferretti 2020). Besides wild boar, the area hosts two more species of wild ungulates, fallow deer (*Dama dama*) and roe deer (*Capreolus capreolus*). Wolf (*Canis lupus*) is the main predator in the area (Ferretti et al. 2021), and its diet is mainly constituted by wild boar (∼43%–55% occurrence in the diet; ∼33%–39% volume in the diet; Ferretti et al. 2019; Lazzeri et al. 2024). For further details, see Sforzi et al. (2012) and Melini et al. (2019).

### 2.2 Wild Boar Data

Data on wild boar body mass were obtained from animals collected in the framework of population control operations carried out by the MRP Authority to limit the ecological and economic impacts of this ungulate. To optimize the efficiency of control operations, they were realized through trapping and removal and selective culling through shooting by Park rangers (by stalking and/or fixed locations). The first method occurred using up to ∼10 fixed corral traps/year and up to ∼13 mobile traps/year, baited with wet maize. Locations of mobile traps changed opportunistically, according to the evidence of wild boar presence in areas sensitive to crop damage. Once culled/trapped, individuals were classified into four age classes based on morphology, body development and proportions, and coat color, further verified by tooth eruption (Matschke 1967). Wild boar were assigned to three age categories (Servanty et al. 2009), namely “piglets” (0–6 months), “juveniles” (6 to <12 months), “yearlings” (12–24 months), and “adults” (over 24 months). Since the main focus was to investigate body mass variations during the harsher season, we selected data from wild boar harvested from June to September, as well as from years, sexes, and age classes with greater consistency in harvest strategy across years. Since our hypotheses focused on the life cycle of yearling and adult individuals, only wild boar classified as belonging to these age classes were included in the analyses. Uncertain cases as well as “piglets” and “juveniles” were excluded. Management aims changed according to variations in wild boar densities; therefore, quotas for trapping/culling were not constant across the years. Data on male body mass came from culling conducted in summer from 2007 to 2022 (627 individuals), whereas data on female body mass came from trapping conducted in summers from 2013 to 2017 (119 individuals). Body mass was measured through an electronic scale with an accuracy of ±1 kg. Males were shot and weighed after evisceration, meaning their body mass was referred to as eviscerated carcasses. Females were trapped and translocated alive; thus, their body mass corresponded to their full weight. We acknowledge a limitation in our approach as using full weight in the female analysis may introduce some variability not directly related to actual body condition (e.g., recent feeding or physiological state). However, our objective was to assess inter‐annual and intra‐sexual variations in body mass during the harsher season in absolute terms. By maintaining the same methodology (i.e., study area, period, and weighing method) across years, we ensured that any potential bias within the female sex analysis remained constant, allowing for temporal comparisons. Furthermore, since we aimed to understand the drivers of body mass in absolute terms, standardizing body mass by body size to obtain body condition indices (e.g., Briedermann 1990; Cellina 2008) was unnecessary, as body mass is positively and strongly correlated with several morphometrics measurements in wild boar (Baruzzi et al. 2023), which would allow comparisons over time.

Data on wild boar density were obtained from regular population monitoring conducted in summer (i.e., post‐reproductive density) since 2007 through feces counts, whose sampling design has been repeatedly described (e.g., Fattorini et al. 2011; Ferretti et al. 2023).

### 2.3 Meteorological and Land Use Data

For each sampled individual, the cumulative rainfall (mm) and average temperature (°C) over the three months preceding the weighing date were calculated. Three months were selected as the timespan conservatively reflecting the effect of accumulated temperatures and rainfall on vegetation productivity (0.6 to 2.8 months, depending on vegetation type; Ding et al. 2020), while still including the physiological time needed for wild boar to convert food resources in body mass. Weather data were gathered from the available professional meteorological station closest to the study area (managed by the Servizio Idrologico Regione Toscana; https://www.sir.toscana.it) as an indicator of relative climatic variation over the study period. The San Donato station (ID: TOS03003099; ∼6 km far from the MRP boundaries and 15 km far from the MRP centroid) worked for the 2007–2011 period while the Alberese station (ID: TOS11000103; inside the MRP, ∼ 6 km far from the centroid) covered the period 2012–2021. Other weather stations are further away from the study area and were not considered to be representative of meteorological conditions experienced by wild boar in the MRP.

The Mediterranean climate during summer (June 21–September 21) is typically characterized by high temperatures, which gradually increase as the season progresses, peaking in July–August (Figure S1). Consequently, the 3‐month accumulated temperature is positively and strongly correlated with the date, that is, the day of the year (*r* = 0.97 for females and *r* = 0.93 for males). The primary aim of this study was to assess drivers of wild boar body mass variations across summers. Therefore, it was crucial to account for date in the models and identify the seasonal effect. To allow accounting for the effects of both temperature and rainfall variations, these meteorological variables were incorporated into a single metric of aridity, the Gaussen aridity index (GI; Gaussen 1954), which was not collinear with the progressive day of the year (*r* = −0.33 for females and *r* = −0.23 for males). This index has been used consistently in studies on the population dynamics of Mediterranean ungulates (Toïgo et al. 2006), including the wild boar (Imperio et al. 2012). It was computed as the 3‐month cumulated rainfall (in mm) minus twice the 3‐month mean temperature (in °C). High GI values correspond to wet conditions accumulated over the previous months, while low values indicate dry and/or drought conditions, reflecting the severity of summer drought and serving as a proxy for resource availability (Imperio et al. 2012).

Trapping sites were georeferenced, whereas culling locations were not; hence, land use effects could be considered only for the analyses on females. The land cover classification was obtained by updating the CORINE Land Cover layer (CLC) provided by the Tuscany Region (reference year: 2019, improving the CLC version 2018; https://www502.regione.toscana.it/geoscopio) through personal observations *in situ*. Since the MRP boundaries are not fenced, land use was considered both inside and in a 1.1‐km buffer outside the Park. The size of this additional zone was derived as the mean of annual home ranges (weighted by number of individuals sampled) of wild boar females calculated by Massei et al. (1997) in our study area (∼3.8 km^2^; radius: ∼1.1 km). Following the same criterion, to identify the main habitats exploited by the trapped females, the QGIS 3.32 buffer feature was used to derive the percentage of land use classes in circular plots embracing a radius of 1.1 km around each trapping site, as previously done (Calosi et al. 2024).

### 2.4 Statistical Analyses

We run separate model sets, one for each sex, stratifying the models by major age classes to account for relevant variability in body mass.

#### 2.4.1 Males

Before fitting the model, the collinearity among covariates was assessed. No significant collinearity was detected among the remaining explanatory variables (all Pearson correlation coefficients *r* were below |0.5|). We performed statistical analyses through linear models (LMs). The response variable was the body mass (kg) of each adult or yearling wild boar male harvested in the study period (eviscerated carcass mass), which was ln‐transformed to improve model residuals. The model included the following fixed effects: (1) date (continuous, as the progressive day of the year); (2) 3‐month GI before the individual weighting date (continuous, as Gaussen aridity index values); (3) age class (categorical; reference level: yearling); (4) population density (continuous, as individuals/km^2^); the interactive effects between (5) the progressive day of the year and 3‐month GI, to evaluate whether the effect of summer progression on body mass depended on drought severity; and between (6) the progressive day of the year and population density, to assess whether the effect of summer progression on body mass depended on density. The following interactions were also included: (7) the 3‐month GI and density, to investigate whether the effect of drought on body mass was mediated by density; (8) age class and population density; (9) age class and progressive day of the year; (10) age class and 3‐month GI, to assess whether the effects of these environmental variables differed between age classes. Insights on landscape drivers were not investigated for males because culling operations were not geo‐referenced.

#### 2.4.2 Females

Consistent with the model for males, collinearity among covariates was assessed before fitting the model. Wild boar population density was collinear with the 3‐month GI (*r* = 0.61). Given the crucial role of rainfall and temperatures in enhancing food resource availability, and since our goal was to assess the effects of weather changes on body mass during summer over the years, population density was removed and 3‐month GI was retained. However, “Year” was included as a random intercept to account for potential inter‐annual variations of body weight associated with density. No significant collinearity was detected among the remaining explanatory variables (all *r* < |0.5|). Statistical analyses were performed through linear mixed models (LMMs). The response variable was the full body mass (kg), which was ln‐transformed to improve model residuals. The model included the following fixed effects: (1) date (continuous, as the progressive day of the year); (2) 3‐month GI before the individual weighting date (continuous, as Gaussen aridity index values); (3) age class (categorical; reference level: yearlings); the cover percentages of (4) ecotonal habitats, (5) agricultural land, and (6) shrubwood/oakwood, all calculated within the 1.1‐km circular buffer (continuous, as %); the interactive effects between (7) progressive day of the year and 3‐month GI, (8) age class and progressive day of the year, and (9) age class and 3‐month GI, to assess whether the effects of these environmental variables differed between age classes.

#### 2.4.3 Model Selection

In both models, covariates were scaled to improve model convergence and interpretability of interactive effects, as well as to allow comparison of effect sizes across different predictors. There was no multicollinearity among explanatory variables for each global model as all the variance inflation factors (VIFs) checked through the R package “performance” (Lüdecke et al. 2021) were below 2.3. Since each possible combination of predictors could represent a different *a priori* hypothesis that could not be discarded in advance, an all‐subset model selection was carried out from both full models (Harrison et al. 2018). Hence, all the possible combinations among predictors (also including the null model) were ranked and weighted from each full model. The AICc and ΔAICc (i.e., the AICc difference between the model and the model with the lowest AICc) values of each model were considered for assessing the model ranking. Following the “nesting rule” (Harrison et al. 2018), the models with ΔAICc < 2 were selected and, among those, only the models that were simpler alternatives of models (if any) with a lower AICc value were retained, to avoid selecting overly complex models. Model weight was standardized within the subset of selected models. A single selected model was obtained from each full model. The coefficients of predictors and 95% confidence intervals were estimated from the best model. The effects of predictors were assessed by checking whether the 95% confidence intervals of coefficients overlapped with zero. The best models were validated by visually checking residual patterns (Zuur et al. 2009). Model fit was assessed using the adjusted‐*R* ^2^ for the LM, and conditional pseudo‐*R* ^2^ for the LMM (Nakagawa et al. 2017). The modeling and model selection were performed using the R packages “glmmTMB” (Brooks et al. 2017) and “MuMIn” (Bartoń 2023), respectively.

## 3 Results

### 3.1 Males

Only one model was selected on factors influencing male body mass (Table 1). This model included the effects of age class, population density, progressive day of the year, 3‐month GI, and the interaction between density and 3‐month GI (Table 2; adjusted *R* ^2^ = 0.59).

**TABLE 1:** Model selections for factors influencing wild boar body mass variations estimated through a LM (a: males) and a LMM (b: females). The model on female body mass included the year as a random effect. The selected model as well as the null model are shown for each analysis, together with their number of parameters (*K*), log Likelihood (LogLik), AICc, ∆AICc, and weight.

Response variable	Model	Variables	*K*	LogLik	AICc	∆AICc	Weight
a. Male body mass	Best	Age class + density + day of the year + 3‐month Gaussen aridity index + density × 3‐month Gaussen aridity index	**7**	−**71.1**	**156.5**	**0.0**	**1**
	Null model		0	−346.1	696.3	539.8	—
b. Female body mass	Best	Age class + ecotone %+ day of the year + 3‐month Gaussen aridity index + age class × 3‐month Gaussen aridity index + day of the year × 3‐month Gaussen aridity index	**9**	**74.5**	−**129.3**	**0.0**	**1**
	Null model		1	43.9	−81.9	47.4	—

**TABLE 2:** Parameters estimated from the top‐ranked LM (a: males) and LMM (b: females) predicting wild boar body mass: coefficients (B) and 95% confidence intervals (95% CIs). An asterisk marks the coefficients whose 95% CIs do not include 0. The reference category for “Age class” is Yearling. Variance of random intercepts in the model (b) (σ^2^) is also shown.

Model	Predictor	*B* coefficient	95% CIs
a. Males	Intercept	4.019	3.993; 4.046*
	Day of the year	0.025	0.002; 0.048*
	Age class [Yearling]	−0.675	−0.722; −0.627*
	3‐month Gaussen aridity index	0.043	0.020; 0.067*
	Density	−0.048	−0.071; −0.025*
	Density × 3‐month Gaussen aridity index	0.039	0.015; 0.062*
b. Females	Intercept	3.626	3.588; 3.665*
σ^2^ = 0.021	Day of the year	0.012	−0.015; 0.039
	Age class [Yearling]	−0.200	−0.251; −0.148*
	3‐month Gaussen aridity index	0.056	0.019; 0.094*
	% Ecotone	0.037	0.014; 0.061*
	Day of the year×3‐month Gaussen aridity index	0.048	0.017; 0.078*
	3‐month Gaussen aridity index×Age class	−0.060	−0.116; −0.005*

**FIGURE 2:**
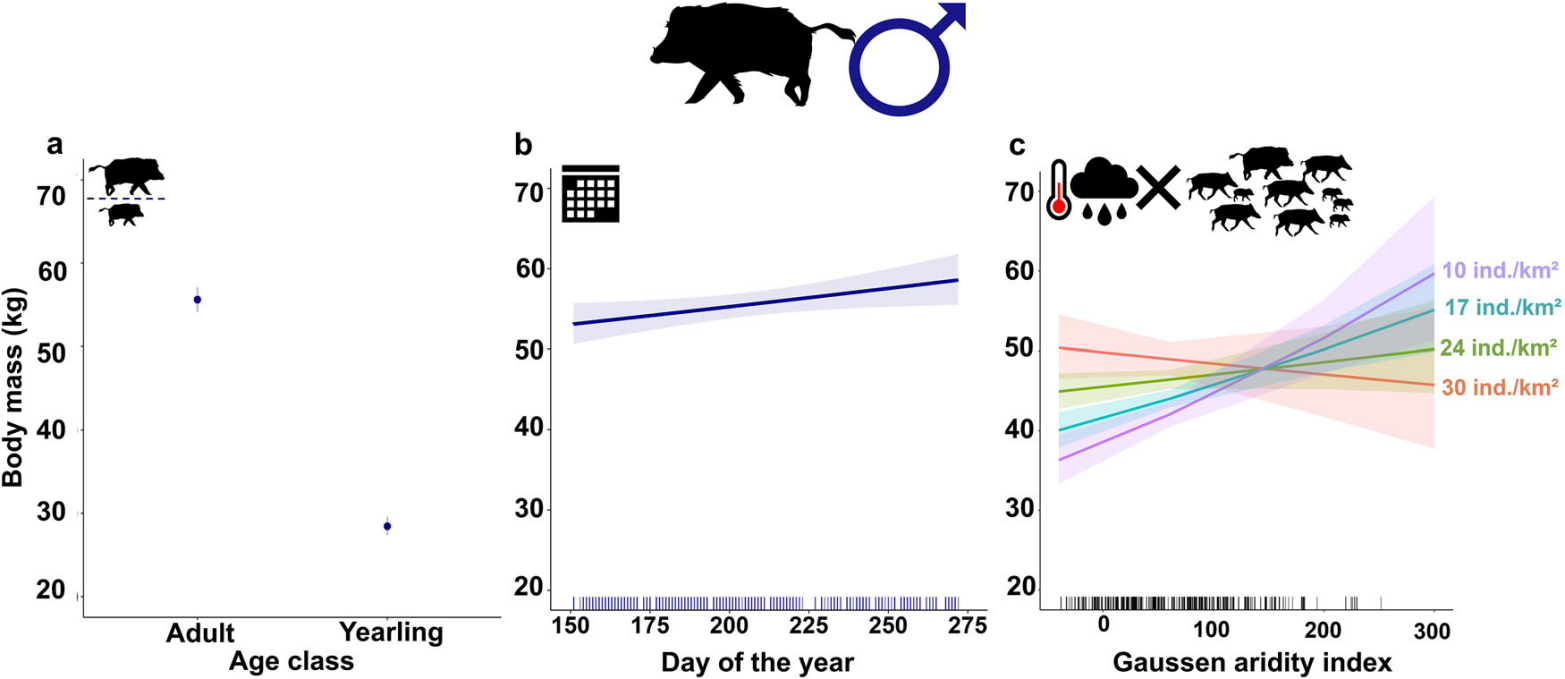
Results for males. Relationships between male eviscerated body mass and (a) age class (adult/yearling), (b) progressive day of the year, and (c) the interaction between density and 3‐month Gaussen aridity index, estimated by the LM. The marks along the *x*‐axis show the distribution of the observed values for each covariate. Lines: intervals predicted values for each covariate. Bands: 95% confidence intervals.

The results showed that body mass (i) was higher in adults than in yearlings (Figure 2a); (ii) increased throughout summer (Table 2; Figure 2b); (iii) decreased with increasing population density (Figure 2c); (iv) increased with the increase of 3‐month GI before the harvest, but only in years with lower population density, whereas the positive effects of rainy seasons disappeared in years with higher density (Table 2; Figure 2c).

### 3.2 Females

**FIGURE 3:**
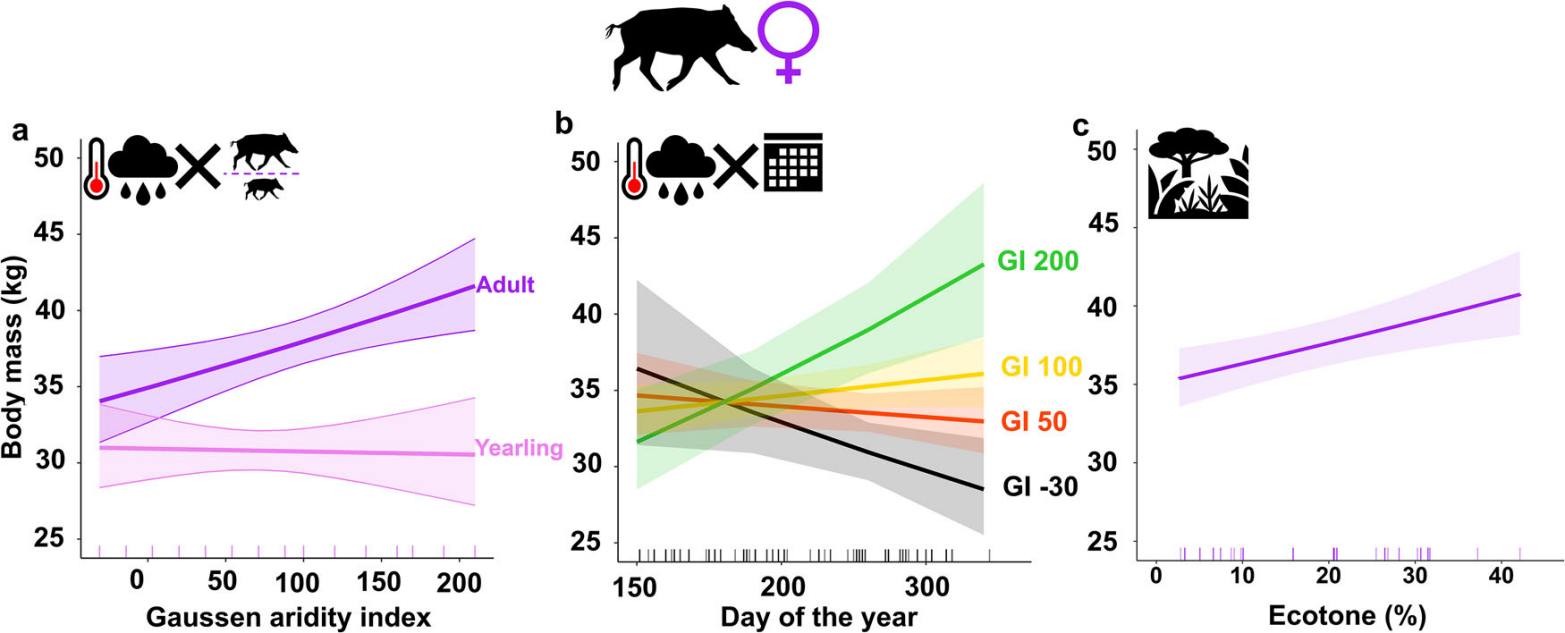
Results for females. Relationships between female body mass (full weight) and (a) the interaction between age class and 3‐month Gaussen aridity index (GI), (b) the interaction between GI and the progressive day of the year, and (c) % ecotone in the buffer, estimated by the LMM. The marks along the *x*‐axis show the distribution of the observed values for each covariate. Lines: predicted values for each covariate. Bands: 95% confidence intervals. GI aridity levels: 200—very low; 100—low; 50—moderate; −30—severe.

Only one model was selected on factors influencing female body mass (Table 1). It included age class, the percentage of ecotone habitats around the trapping location, the progressive day of the year, the 3‐month GI, the interaction between age class and 3‐month GI, as well as the interaction between the day of the year and 3‐month GI (Table 2; pseudo‐*R* ^2^ = 0.48). Body mass (i) was higher in adults than in yearlings (Figure 3a) and (ii) did not change throughout summer when the marginal effect of date was considered; rather, body mass variation throughout summer was modulated by aridity: body mass increased with the summer progression in rainy‐mild years, decreased in hot‐drier years and did not change in “average” years (Figure 3b). Body mass (iii) generally increased with the GI in the 3 months before the harvest (i.e., with rainy‐mild weather conditions; Figure 3a,b). However, the effects of summer aridity differed between age classes: rainy‐mild weather favored adult female body mass, but did not affect yearling mass (Figure 3a). Body mass (iv) increased in sites with higher ecotone cover included in the buffer (Figure 3c).

## 4 Discussion

We analyzed summer variations of wild boar body mass in a Mediterranean area, where hot‐dry weather conditions make this season particularly limiting for wild ungulates (Massei et al. 1997, 1996). In males, summer aridity and density interacted in affecting body mass, with the positive effect of “rainy‐mild” conditions disappearing in years with high density. In females, mass gains were reported in “rainy‐mild”’ years and mass losses occurred in “hot‐dry” years.

For ungulates, body mass is a critical factor in enhancing individual survival during harsh seasons (Mysterud and Sӕther 2011). Heavier individuals can afford food scarcity for longer periods than lighter ones, thanks to greater fat reserves (Calder 1984; Mysterud and Sӕther 2011). During summer, adult females generally face the costs of lactation and other maternal care (Spitz et al. 1998; Apollonio et al. 2011; Scandura et al. 2022). This, combined with the scarcity of natural food resources, may result in increasing energy expenditure (Albon et al. 2017; Ruf et al. 2021; Scandura et al. 2022). Consequently, females may deplete their fat reserves to cope with resource limitations, leading to body mass loss (Mauget 1982; Trondrud et al. 2021). During the same period, males are generally solitary and adopt compensatory strategies to mitigate seasonal challenges (Scandura et al. 2022). They can reduce their mobility (Massei et al. 1996), increase nocturnal activity (Podgórski et al. 2013; Ruf et al. 2023), and select habitats for their thermal shelter potential as well as the proximity to high‐quality food resources (Ruf et al. 2023). These strategies allow males to maximize food intake and minimize energy expenditure (Ruf et al. 2023), thereby limiting fat reserve depletion.

Nevertheless, in this study, wild boar body mass did not decrease generally throughout summer for both sexes, not supporting our prediction (1). Mass variations of adult females were modulated by cooler and rainy summers, ranging from a seasonal decrease to an increase along a gradient of decreasing aridity. Males showed a mass gain throughout the summer. These results indicate intra‐sexual and inter‐annual variations in the resource allocation in body mass modulated by environmental variation and suggest that in cooler‐rainy years wild boar could get adequate food resources, limiting the necessity to erode fat reserves.

We showed an increase in female body mass with increasing GI in the 3 months before the harvest, confirming our prediction (2). In males, this effect was modulated by population densities, disappearing in years with higher densities. Intra‐ and inter‐annual climatic fluctuations characterize Mediterranean environments; the interaction between them and the other drivers on body mass variations may buffer or increase the effect of single factors (Mysterud and Sӕther 2011). The effect of summer progression was mediated by cooler‐rainy summers. Water availability is expected to be a major limiting factor in the southern distribution range of most ungulates (Mysterud and Sӕther 2011), especially in climates with prolonged drought such as the Mediterranean ones (Giralt‐Rueda and Santamarίa 2023). By disentangling body mass variations of females across years, we found a gain in body mass in cooler‐rainy years, a mass loss in hotter‐drier years, and no change in “average” years. Higher humidity and precipitation could directly affect vegetation growth (Giralt‐Rueda and Santamarίa 2023), increasing the availability of food resources both above and underground (Hone 1995). Moisture also makes the soil easier to dig (Welander 2000; Sandom et al. 2013) and favors the efficiency of wild boar sense of smell (Brivio et al. 2017), while facilitating the rooting activity (Calosi et al. 2024). In turn, rainfall and lower temperatures, in spring‐summer, should improve foraging conditions, prompting stability or increase in body mass (Colomer et al. 2024, for wild boar; see also Vannini et al. 2021; Giralt‐Rueda and Santamarίa 2023, for deer species).

Density‐dependent variations in body mass have been frequently reported among ungulates (Festa‐Bianchet et al. 1998; Toïgo et al. 2006; Morellet et al. 2007; Bonardi et al. 2016). We confirmed this trend in wild boar males, for which population density could also modify the effects of weather on body mass, acting as a potential key factor in buffering the impact of climate on individual survival (Fryxell and Sinclair 1988; Gordon and Illus 1989; Colomer et al. 2024). The study area is bordered by the sea on its western side and is surrounded by anthropical barriers (e.g., towns, railway, highway) along most other borderlines. This landscape configuration implies a shortage of sites potentially suitable for movements outside the area following changes in food availability. Furthermore, most of the study area (∼55%) is covered by habitats that offer a short range of suitable food during summer, such as Mediterranean sclerophyllic shrubland, pinewood, and dry wetlands (Spitz et al. 1998; Torres‐Porras et al. 2015), as well as human settlements and seaside. Areas with the greatest foraging opportunities are concentrated in ecotones and active crops in the agricultural area. Although higher rainfall is expected to increase plant productivity, thus enhancing food availability, this increase could not be generally enough to sustain the energetic requirements of high wild boar numbers, thus leading to a reduced allocation in individual body mass in years with the highest densities and the lower rainfall. We showed an increase in female body mass with rising ecotone availability, partially supporting our prediction (3). These habitats include transitional sites between concealed habitats and open fields or agricultural areas and are usually selected by wild ungulates because they provide the best trade‐off between sheltering from stressors (e.g., humans, predators, and heat), and the availability of high‐quality food (McLoughlin et al. 2007; Miyashita et al. 2008; Laguna et al. 2021). In our study area, during summer, wild boar have been reported to use ecotones three times more than the other habitat types (Fattorini and Ferretti 2020). Furthermore, in the MRP, ecotone areas also host the habitat type identified by the Natura2000 code 6220 “Pseudo steppe with grasses and annuals (*Thero‐Brachypodietea*),” protected under the EU Habitat Directive (Labadessa et al. 2023). This habitat includes herbaceous plant communities adapted to dry conditions and is particularly attractive for wild boar (Calosi et al. 2024). The availability of grasslands may play a role in maintaining/improving wild boar body condition in summer during rainy years, when water availability may induce greater and faster vegetation growth.

In our study area, summer is the most limiting season for wild ungulates and is usually characterized by reduced availability of natural food resources (Massei et al. 1997, 1996; Minder 2012). The absence of mass loss during the most limiting season, except for drier years in females, indicates that wild boar take advantage of alternative food resources. Agricultural lands are sources of additional energetic food supply for wild ungulates, especially in summer, potentially enhancing their body growth (Hewison and Gaillard 2001, for roe deer; Vannini et al. 2021, for red deer *Cervus elaphus*; Gethöffer et al. 2023, for wild boar). While a general avoidance of cultivated areas has been reported in agroforestry lands (Thurfjell et al. 2009; Jánoska et al. 2018; Laguna et al. 2021), wild boar can be attracted by active crops, especially when they are located near forests or ecotones and in years with a lack of natural food resources (Schley and Roper 2003; Herrero et al. 2006; Ficetola et al. 2014; Laguna et al. 2021). The model did not provide support for the effect of agricultural land percentage within the buffer on female body mass. A previous study in the same area showed that agricultural land as a whole was used less than ecotonal habitats and comparably with shrubwood and pinewood, in summer (Fattorini and Ferretti 2020). In our study area, agricultural land includes heterogeneous habitats, with fields that likely differ in attractiveness to wild boar, ranging from active (thus attractive) to plowed/harvested (therefore unattractive) crops (Fattorini and Ferretti 2020; Ferretti et al. 2021). Wild boar are expected to use fields with available crops (Keuling et al. 2009; Thurfjell et al. 2009). An average of 39.7 damage events/year were reported in the study area in 2010–2023 (range: 18–63, mainly wheat, cereals, and sunflowers), although with a decreasing trend throughout the last 12 years due to population control and preventive measures (MRP Agency archive). Thus, we suggest that the effect of the use of crops on wild boar mass variation in summer cannot be ruled out. Probably, cultivated crops are reached by wild boar through rapid movements at short timescales (e.g., at nighttime), during critical maturation stages of focal crops, and beyond the typical home range scale, which may have led to an underestimation of the use of crops over other habitats (Fattorini and Ferretti 2020; Ferretti et al. 2021). Studies based on wild boar movements would be necessary to confirm such a hypothesis (Keuling et al. 2009; Thurfjell et al. 2009). If confirmed, these results would suggest that access to high‐quality and energetic food resources, such as those provided by crops, could sustain wild boar body mass during periods when the availability of natural food resources is scarce, for example, in summer. Further insights can ensue for the management of wild boar populations in anthropized landscapes to mitigate human–wildlife conflicts. For instance, this emphasizes the importance of limiting the access of these ungulates to cultivations through preventive measures such as appropriate fences.

This study is subject to some limitations, such as the small sample size of females. A wider female sample size would have allowed us to better understand the effect of weakly influential variables. Additionally, standardizing individual body mass by body size measurements would have improved knowledge on individual resource allocation into body condition and inter‐age variation, despite most body size measurements strongly predict body mass in wild boar (Baruzzi et al. 2023). Eventually, the differences in weighing methods between sexes may have influenced the interpretation of inter‐sexual differences. Our aim was to evaluate which factors drive body mass fluctuations within sexes, and in absolute terms, therefore, our conclusions are expected not to be affected by these issues. Nonetheless, further analyses based on larger sample size, consistent weighing methods, and body size‐standardized measurements may improve our findings, especially those regarding sex and age class differences.

We showed how the complex interplay of environmental factors such as seasonality, weather, land use, and population density contributed to shape variations in wild boar body mass in a Mediterranean area. This suid is an omnivorous, flexible, and adaptive ungulate occurring in several habitats and biogeographic zones worldwide (Scandura et al. 2022). In the last decades, wild boar have raised their densities and distribution, thanks to the combination of several concomitant factors. These include the increase of suitable habitats promoted by the abandonment of extensive agricultural activities, deliberate releases, combined with species‐specific biological factors (e.g., high reproductive rates and dispersal potential), as well as milder winters (Massei et al. 2015; Vetter et al. 2015; Markov et al. 2022, 2019). Considering the wild boar global distribution, limiting factors may differ along biogeographic regions. Winter severity may affect individuals in the northernmost eco‐zones (Thurfjell et al. 2014), food scarcity may limit wild boar in central zones with milder climates (Gethöffer et al. 2023), while drought is crucial in Mediterranean eco‐zones (Massei et al. 1997, 1996; this study). It has been shown that temperature increase could facilitate wild boar populations at higher latitudes and in mountainous areas, mainly by mitigating winter severity (Geisser and Reyer 2005; Vetter et al. 2020, 2015; Brogi et al. 2023). Nevertheless, the same increase at southern latitudes could be detrimental, because high temperatures are usually correlated with drought, which in turn could limit individual body mass, and thus health and reproductive success (Massei et al. 1996; Fernández‐Llario and Mateos‐Quesada 1998; Fernández‐Llario et al. 1999; this study). Demographic and population effects are mostly linked to the survival of piglets and juveniles younger than 12 months (González‐Crespo et al. 2018). Thus, the loss of body condition by females in summer could affect the survival of their dependent piglets, either through decreased lactation or through exposure to the same food shortage conditions, with significant effects on population dynamics (González‐Crespo et al. 2018). Additionally, the present results suggest that, in a Mediterranean area characterized by sclerophyllic forests, grasslands, and agricultural landscapes, wild boar may be buffered against the summer harshness and may maintain—or even increase—body mass during the major limiting season. The role of cultivations should be hence clarified, and, if confirmed to occur broadly across Mediterranean regions, it would highlight the contribution of anthropogenic activities in influencing key drivers of population dynamics of this expanding, generalist mammal. Management actions aimed at controlling wild boar densities and impacts should account for local characteristics and weather variations. As a gregarious species, the wild boar tends to form large family groups, particularly in summer when females are weaning their offspring (Massei et al. 1997; Barrios‐Garcia and Ballari 2012; Scandura et al. 2022). This clustering may amplify behaviors such as rooting and trampling, locally intensifying their impact on soil and vegetation in the foraging sites (Massei et al. 1997; Hone 2002; Barrios‐Garcia and Ballari 2012). During dry years, the shortage of foraging sites could further concentrate these effects, raising concerns when they overlap with habitats of conservation interest, such as those protected under the EU Habitat Directive (e.g., habitat “6220”; Calosi et al. 2024), or with agricultural areas (Herrero et al. 2006). As a result, dissuasive actions as well as population control activities should be enhanced in dry years and focused in proximity to attractive contexts, that is, locations with greater foraging opportunities.

## Author Contributions

**Martina Calosi**: data curation, formal analysis, writing – original drafts; **Niccolò Fattorini**: formal analysis, writing – original drafts; **Andrea Sforzi**: data curation, writing – original drafts; **Luca Tonini**: data curation; **Francesco Ferretti**: conceptualization, data curation, formal analysis, supervision, writing – original drafts.

## Supporting information




**Figure S1** Mean temperature variations in summer (i.e., from the 21^st^ of June to the 21^st^ of September) for each sampled year. Months in the x axis are the following: 6, June; 7, July; 8, August; 9, September.
