# Supplementary material for: Starving or Stuffing? Plasticity in Wild Boar Body Mass Variations During Summer in a Mediterranean Area
Source: Integr Zool. 2025 Aug 6;21(3):577–88. doi: 10.1111/1749-4877.13012 (PMC13164837; doi:10.1111/1749-4877.13012)
Supplement: Supplementary file 1 — Figure S1 Mean temperature variations in summer (i.e., from the 21st of June to the 21st of September) for each sampled year. Months in the x axis are the following: 6, June; 7, July; 8, August; 9, September. [file INZ2-21-577-s001.docx]

**SUPPORTING INFORMATION**

**Figure S1.** Mean temperature variations in summer (i.e., from the 21^st^ of June to the 21^st^ of September) for each sampled year. Months in the x axis are the following: 6, June; 7, July; 8, August; 9, September.
